# ABSCISIC ACID-INSENSITIVE 4 negatively regulates flowering through directly promoting Arabidopsis *FLOWERING LOCUS C* transcription

**DOI:** 10.1093/jxb/erv459

**Published:** 2015-10-27

**Authors:** Kai Shu, Qian Chen, Yaorong Wu, Ruijun Liu, Huawei Zhang, Shengfu Wang, Sanyuan Tang, Wenyu Yang, Qi Xie

**Affiliations:** ^1^State Key Laboratory of Plant Genomics, National Center for Plant Gene Research, Institute of Genetics and Developmental Biology, Chinese Academy of Sciences, Beijing 100101, PR China; ^2^Key Laboratory of Crop Ecophysiology and Farming System in Southwest China, Institute of Ecological Agriculture, Sichuan Agricultural University, Chengdu 611130,PR China; ^3^University of Chinese Academy of Sciences, Beijing 100049, PR China

**Keywords:** ABA, ABI4, chromatin immunoprecipitation, FLC, flowering, transcription factor

## Abstract

During the life cycle of a plant, one of the major biological processes is the transition from the vegetative to the reproductive stage. In *Arabidopsis*, flowering time is precisely controlled by extensive environmental and internal cues. Gibberellins (GAs) promote flowering, while abscisic acid (ABA) is considered as a flowering suppressor. However, the detailed mechanism through which ABA inhibits the floral transition is poorly understood. Here, we report that ABSCISIC ACID-INSENSITIVE 4 (ABI4), a key component in the ABA signalling pathway, negatively regulates floral transition by directly promoting *FLOWERING LOCUS C* (*FLC*) transcription. The *abi4* mutant showed the early flowering phenotype whereas *ABI4*-overexpressing (*OE-ABI4*) plants had delayed floral transition. Consistently, quantitative reverse transcription–PCR (qRT–PCR) assay revealed that the *FLC* transcription level was down-regulated in *abi4*, but up-regulated in *OE-ABI4*. The change in *FT* level was consistent with the pattern of *FLC* expression. Chromatin immunoprecipitation-qPCR (ChIP-qPCR), electrophoretic mobility shift assay (EMSA), and tobacco transient expression analysis showed that ABI4 promotes *FLC* expression by directly binding to its promoter. Genetic analysis demonstrated that *OE-ABI4::flc-3* could not alter the *flc-3* phenotype. *OE-FLC::abi4* showed a markedly delayed flowering phenotype, which mimicked *OE-FLC::WT*, and suggested that *ABI4* acts upstream of *FLC* in the same genetic pathway. Taken together, these findings suggest that ABA inhibits the floral transition by activating *FLC* transcription through ABI4.

## Highlight

*FLC* is the direct target of both of the transcription factors ABI4 and ABI5, and ABA inhibits floral transition by activating *FLC* transcription through ABI4.

## Introduction

As sessile organisms, plants monitor the changes in both external and internal signals, including photoperiod, temperature, and phytohormonal levels, to decide their flowering initiation (Andres and Coupland, 2012; Song *et al.*, 2013). This transition from vegetative to reproductive growth is one of the major developmental phases during the life cycle of a plant (Boss *et al.*, 2004; He, 2012). Optimizing flowering time is crucial to reproductive success, and hence is of great agricultural value, particularly when one considers the issues posed by climate change (Craufurd and Wheeler, 2009; Michaels, 2009; Riboni *et al.*, 2013). Subsequently, plants have evolved diverse cryptic mechanisms to regulate the timing of flowering precisely.

The distinctive regulatory mechanisms comprising the photoperiod-, autonomous-, vernalization-, and gibberellic acid (GA)-dependent pathways have already been fully elucidated in *Arabidopsis* (He, 2012). These four pathways form a regulatory network that enables plants to integrate endogenous developmental signals with responses to environmental inputs, including daylength, light quality, and temperature. In this complicated network, the MADS box-containing transcription factor gene *FLOWERING LOCUS C* (*FLC*) is a potent integrator, which negatively regulates flowering initiation (Michaels, 2009; Michaels and Amasino, 1999; Son *et al.*, 2014; Berry and Dean, 2015). Consequently, overexpression of *FLC* results in a late-flowering phenotype (Hepworth *et al.*, 2002). Generally, *FLC* expression is silenced by vernalization treatment and the autonomous pathway. This involves histone methylation and change of chromatin structure (Bastow *et al.*, 2004; Michaels, 2009). As a transcription factor, FLC represses the expression of *SUPPRESSOR OF OVEREXPRESSION OF CONSTANS 1* (*SOC1*), *FLOWERING LOCUS D* (*FD*), and *FLOWERING LOCUS T* (*FT*) through directly binding to the promoter regions of *SOC1* and *FD* or the first intron of *FT*. Thus these three genes represent further key factors in regulating flowering time (Searle *et al.*, 2006). In particular, *FT* encodes ﬂorigen, a ﬂowering inducer (Corbesier *et al.*, 2007). *FLC* is a key repressor in the initiation of flowering and links the diverse flowering time regulatory pathways (Searle *et al.*, 2006; He, 2009, 2012; Angel *et al.*, 2015). Although numerous studies have investigated the diverse components downstream of transcription factor FLC, including *SOC1* and *FD*, the key regulators that act upstream of FLC remain elusive. A better understanding of these will improve our understanding of FLC-mediated plant floral transition.

Besides the effects of environmental cues on plant floral transition, internal phytohormones also play a key role in this process. An early study revealed that GA stimulates bolting in henbane (*Hyoscyamus niger*) (Lang, 1957). Numerous subsequent studies demonstrated that GA induces flowering through promoting transcription of the floral meristem identity gene *LEAFY* (*LFY*) (Blazquez *et al.*, 1998; Achard *et al.*, 2004), which is a key determinant in plant floral decision processes. Overexpression of *LFY* in transgenic plants rescues the dramatic delayed-flowering phenotype of the GA-deficient mutant *ga1-3* (Blazquez *et al.*, 1998). The phytohormones abscisic acid (ABA) and GA are the primary endogenous factors which regulate diverse physiological processes antagonistically, including seed germination and plant growth (Seo *et al.*, 2006; Yaish *et al.*, 2010). With regards to floral transition, the antagonistic effect between ABA and GA has also been investigated. The ABA-deficient mutant *aba2* shows the early-flowering phenotype (Domagalska *et al.*, 2010), in contrast to the late-flowering phenotype of the GA-deficient mutant *ga1-3* (Blazquez *et al.*, 1998). In addition, treatment with exogenous ABA delays plant flowering (Wang *et al.*, 2013). Consequently, ABA is considered to be a repressor of plant floral initiation. However, the detailed molecular mechanisms of this are poorly understood.

A pioneering study demonstrated that ABA-delayed flowering may occur in a DELLA-dependent manner (Achard *et al.*, 2006). However, the precise mechanism remains elusive. Recently, an elegant pathway has been described through which ABA affects floral transition negatively (Wang *et al.*, 2013). In this, the transcription factor ABSCISIC ACID-INSENSITIVE MUTANT 5 (ABI5) activates *FLC* transcription by directly binding to its promoter. Thus, ABI5 is an important factor through which ABA inhibits the plant floral transition (Wang *et al.*, 2013). ABI5 is the only regulator found to date that controls plant floral initiation in the ABA signalling transduction pathway. Whether there are others is not known. To date there is little information about the precise mechanisms which underlie ABA inhibition of flowering. Consequently, a better understanding of this will be valuable.

ABSCISIC ACID-INSENSITIVE 4 (ABI4) is an AP2/ERF domain-containing transcription factor and is an enhancer in the ABA signalling pathway that functions particularly during seed development, seed dormancy, and regulation of germination (Finkelstein, 1994; Finkelstein *et al.*, 1998; Söderman *et al.*, 2000; Shu *et al.*, 2013). Numerous elegant studies have demonstrated that *ABI4* is a versatile factor (Wind *et al.*, 2013), which is also involved in many other aspects of plant development, including responses to glucose (Arenas-Huertero *et al.*, 2000), lipid mobilization from the embryo (Penfield *et al.*, 2006), chloroplast and mitochondrial–nucleus retrograde signalling pathways (Koussevitzky *et al.*, 2007; Sun *et al.*, 2011), and plant male sterility (Shu *et al.*, 2014). ABI4 is also involved in the ABA- and jasmonate-dependent signalling cross-talk (Kerchev *et al.*, 2011) and the ABA- and GA cross-talk pathways (Shu *et al.*, 2013). Recently, two independent groups reported that the *abi4* mutant has an early-flowering phenotype, and the two different allele mutants of the *ABI4* locus result in the same phenotype, strongly suggesting that ABI4 has an important role in floral transition (Foyer *et al.*, 2012; Matsoukas *et al.*, 2013). However, the detailed mechanism through which ABI4 regulates flowering initiation remains elusive.

Here, we perform further investigation of the roles of ABI4 in the initiation of flowering. Consistent with previous reports (Foyer *et al.*, 2012; Matsoukas *et al.*, 2013), the *abi4* mutant had the early-flowering phenotype, whereas transgenic overexpression of *ABI4* (*OE-ABI4*) *Arabidopsis* delays the floral transition. Further, we found that the *FLC* expression level was down-regulated in *abi4*, but up-regulated in *OE-ABI4*. Chromatin immunoprecipitation qPCR (ChIP-qPCR), electrophoretic mobility shift assay (EMSA), and tobacco transient expression analysis showed that ABI4 activates *FLC* expression by directly binding to its promoter. Consistent with these results, genetic analysis demonstrated that *OE-ABI4::flc-3* did not alter the *flc-3* phenotype, while *OE-FLC::abi4* changed the *abi4* early-flowering phenotype. This indicates that *ABI4* acts upstream of *FLC* in the same genetic pathway. Taken together, the results of this study suggest that ABI4 is a key factor which negatively regulates flowering through activating *FLC* transcription directly.

## Materials and methods

### Plant materials and growth conditions

*Arabidopsis thaliana* ecotype Columbia-0 was used as the wild type (WT) in this study. The point mutant *abi4-1* (CS8104) was obtained from the Arabidopsis Biological Resource Center (Ohio State University, Columbus, OH, USA). This mutant originated from the Finkelstein laboratory (Finkelstein, 1994; Finkelstein *et al.*, 1998). The plasmid *pro35S::FLC-GFP* was transformed into the *abi4* mutant to generate *OE-FLC::abi4* and, at the same time, *OE-ABI4* was introduced into the *flc-3* mutant background by genetic crossing for the generation of *OE-ABI4::flc-3*. The *abi4* point mutant and the functional *OE-ABI4* lines (OE1 and OE2) had been generated in our previous study (Shu *et al.*, 2013). Using 10% bleach, *Arabidopsis* seeds were surface-sterilized and washed four times with sterile water. The sterile seeds were then suspended in 0.2% agarose and sown on half-strength Murashige and Skoog (1/2 MS) medium. The plates were transferred to tissue culture rooms at 22 ºC under long-day (16h light/8h dark) (LD) or short-day (8h light/16h dark) (SD) photoperiod conditions, depending on the needs of the particular experiments. Ten-day-old seedlings were transplanted into soil and placed in a growth chamber, again under LD or SD conditions, at 22 ºC and 70% relative humidity.

### Generation of transgenic plants

Transgenic plants carrying constitutively expressed *ABI4* which had been generated in a previously reported study (Shu *et al.*, 2013) were also used in this study. In order to produce *OE-FLC::abi4* transgenic plants, the *FLC* coding sequence fragment was amplified by PCR and then cloned into the vector *pCanG-HA-GFP*, in which *FLC* was expressed under the control of the *Cauliflower mosaic virus* (CaMV) 35S promoter. This construct was transformed into the *abi4* mutant background by the vacuum inﬁltration method using the *Agrobacterium tumefaciens* strain EHA105 (Bechtold and Pelletier, 1998). T_2_ seeds were germinated on normal 1/2 MS plates containing 50mg ml^–1^ kanamycin for vector *pCanG-HA-GFP*, and then the resistant seedlings were transferred to soil to obtain homozygous T_3_ seeds. The T_3_ homozygous lines were employed for detailed phenotypic analysis.

### Flowering-time experiment

Plants in a growth chamber (LD or SD conditions) were examined. In this study, flowering time was scored as the days from germination to flowering and the number of total rosette leaves at bolting, according to the protocol of Mai *et al.* (2011). The plants were checked for flower buds every day. Approximately 15–20 plants were examined for each genotype.

### Gene expression analysis

Preparation of total RNA from the 2-week-old seedlings, ﬁrst-strand cDNA synthesis, and quantitative reverse transcription–PCR (qRT–PCR) were performed as previously described (Cui *et al.*, 2012; Shu *et al.*, 2013). The mRNA was subjected to DNase I treatment, and then the total RNA (2 μg) was denatured and employed for reverse transcription using Moloney murine leukaemia virus reverse transcriptase (200U per reaction; Promega Corporation). Quantitative PCR was performed using the CFX96 Touch™ Real-Time PCR Detection System (Bio-Rad) and SsoFast™ EvaGreen Supermix (Bio-Rad). Gene expression levels were quantiﬁed at the logarithmic phase using the expression of the housekeeping *18S* RNA as an internal control. Three biological replicates were performed for each experiment. The primer sequences used for qRT–PCR are shown in Supplementary Table S1 available at *JXB* online.

### Chromatin immunoprecipitation (ChIP)-qPCR assay

ChIP-qPCR assays were performed as previously described (Shu *et al.*, 2013). Transgenic seedlings containing *35S-ABI4-GFP* were harvested (1.5g) on 1/2 MS medium and then cross-linked with 1% formaldehyde. The seedlings were ground in liquid nitrogen, and then the nuclei were isolated. Immunoprecipitation assays were performed with the anti-green fluorescent protein (GFP) antibody and protein G beads. Immunoprecipitation in the absence of anti-GFP served as the control (CK). The DNA was precipitated by isopropanol, and dissolved in water containing 20 μg ml^–1^ RNase. The qPCR analysis was performed using specific primers corresponding to the *FLC* promoter. *TUB4* was used as an internal control. The *ABI5* promoter fragment was used as a positive control since a previous study demonstrated that ABI4 could directly bind to the promoter of *ABI5* (Bossi *et al.*, 2009). The primers used for the ChIP-qPCR assays are shown in Supplementary Table S1 at *JXB* online.

### Analysis of *FLC* promoter activity by ABI4 *in vivo*

The transient expression assay was performed in *Nicotiana benthamiana* leaves as previously described (Yang *et al.*, 2011). The native *FLC* promoter (Pro-*FLC*) was amplified from genomic DNA. This promoter fragment was cloned into the *pCambia1300-221* vector by replacing the original CaMV 35S promoter, and then *pCambia1300-221-ProFLC-GUS* was generated. *pCanG-ABI4-GFP* was the effector construct. The *A. tumefaciens*-mediated tobacco transient transformation was performed according to our previous protocol (Liu *et al.*, 2010). *Agrobacterium* cells were cultured at 28 °C overnight, and then collected and re-suspended with infiltration buffer and infiltrated into tobacco leaves. Based on this, the mutated *FLC* promoters (Supplementary Fig. S4A at *JXB* online) were also generated, in which some key CCAC motif sequences were changed.

β-Glucuronidase (GUS) activity was detected 3 d after infiltration. Leaves were sampled using a hole punch. The total protein was quantified using the Bradford protein assay kit method (Bio-Rad Company, USA). GUS activity for each combination was determined using the protocol described previously with 4-methylumbelliferyl-β-d-glucuronide (Sigma-Aldrich Company, USA) as a substrate (Jefferson *et al.*, 1987). Histochemical staining for GUS was performed according to the method of Stalberg *et al.* (1993). The plant leaves harvested by hole punch were immersed in GUS staining buffer at 37 °C for 16h, and then immersed in 95% (v/v) ethanol to remove the chlorophyll. A Leica MZ16 FA stereomicroscope was used for photography (Leica Company, Germany).

### Electrophoretic mobility shift assay (EMSA)

EMSAs were performed by the Chemiluminescent Nucleic Acid Detection Module (Thermo Company, Product No. 89880), according to a previously published protocol (Wei *et al.*, 2015). The coding sequence of *ABI4* was inserted into the *Bam*HI/*Xba*I sites in the pMalC2 backbone vector which contains a maltose-binding protein (MBP) tag. Then the ABI4 fusion proteins were expressed in *Escherichia coli* (37 °C) and purified. According to the ChIP-qPCR results, we chose the P4 region (60bp, Fig. 3A) as a probe for the EMSA. These single-stranded oligonucleotide sequences were synthesized and then the double-stranded DNA was obtained through heating oligonucleotides at 70 °C for 5min, and annealing in 50mM NaCl solution, then cooling to room temperature. Investigation of the interaction between ABI4 protein and the corresponding probes was carried out according to the protocol provided with the DIG Kit (Roche). The primer sequence for constructing the MBP–ABI4 vector and the probe for the EMSA are given in Supplementary Table S1 at *JXB* online.

## Results

### The early flowering phenotype of the *abi4* mutant

**Fig. 1.**
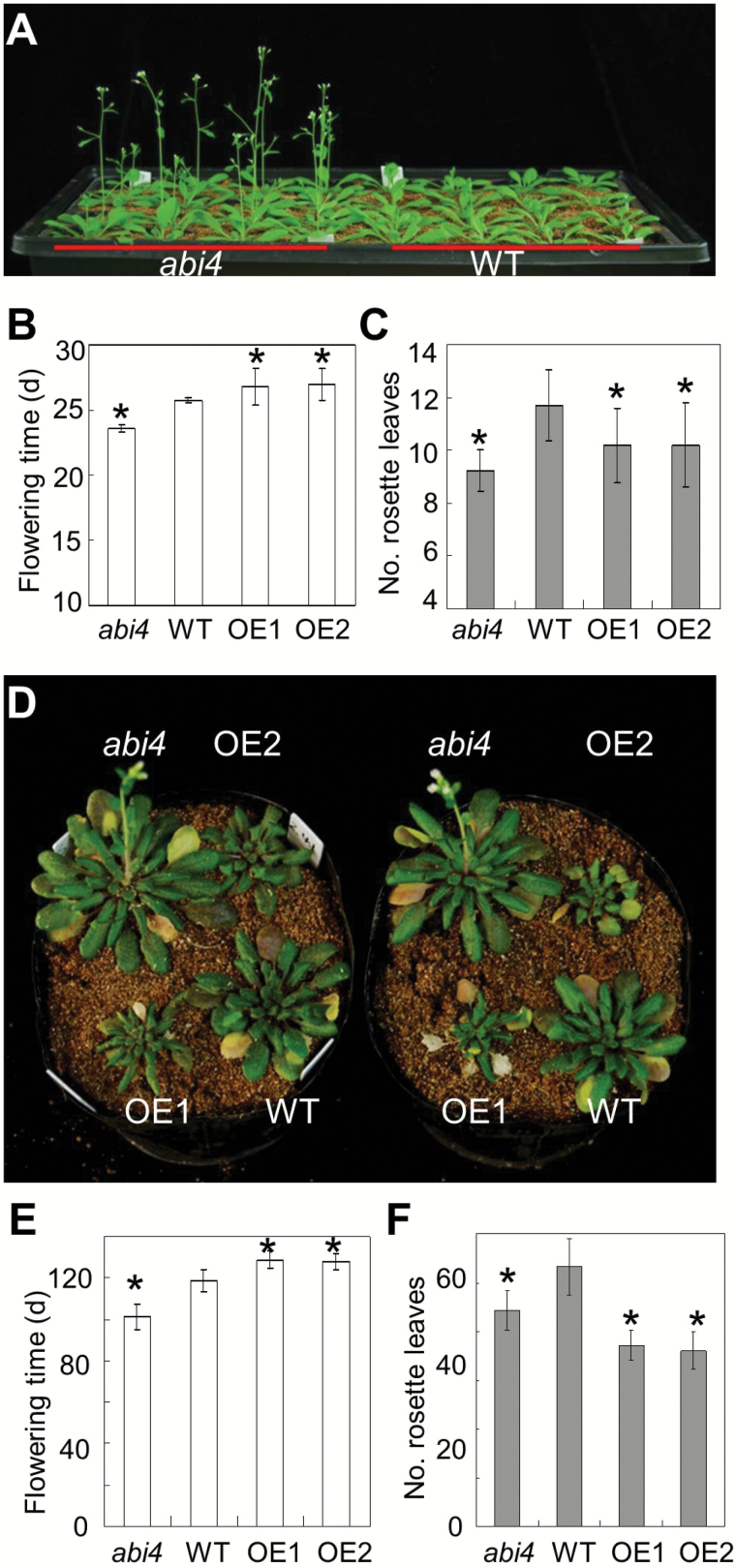
Early-flowering phenotype of *abi4* under long-day (LD) and short-day (SD) growth conditions. (A) Representative (28-day old) *abi4* mutant and wild-type (WT) plants grown under LD conditions. (B) Flowering time scored as the days from germination to bolting of WT, *abi4*, *OE-1*, and *OE-2* genotypes under LD conditions. *n* ≥15; error bars indicate the SE. (C) Flowering time scored as the number of rosette leaves at flowering of WT, *abi4*, *OE-1*, and *OE-2* genotypes under LD conditions. *n* ≥15; error bars indicate the SE. (D) Representative (100-day-old) *abi4*, WT, *OE-1*, and *OE-2* plants grown under SD conditions. (E) Flowering time scored as the days from germination to bolting of WT, *abi4*, *OE-1*, and *OE-2* genotypes under SD conditions. *n* ≥15; error bars indicate the SE. (F) Flowering time scored as the number of rosette leaves at flowering of WT, *abi4*, *OE-1*, and *OE-2* genotypes under SD conditions. *n* ≥15; error bars indicate the SE. An asterisk (*) indicates a significant difference at the *P*<0.05 level by Student’s *t*-test analysis.

In order to assess the effect of *ABI4* on floral initiation control, the flowering time phenotype of *abi4* mutant and transgenic *OE-ABI4* plants was analysed in both LD and SD photoperiods illuminated by white light. *abi4* mutant plants flowered earlier than the WT in both LD (Fig. 1A–C) and SD conditions (Fig. 1D–F), as indicated by the days to flowering (Fig. 1B, E) and the number of rosette leaves (Fig. 1C, F). In contrast, *OE-ABI4* transgenic plants had a late-flowering phenotype when compared with the WT under both LD (Fig. 1B, C; Supplementary Fig. S1 at *JXB* online) and SD (Fig. 1D, E) growth conditions in terms of the days to flowering. However, with regards to the number of rosette leaves, the *OE-ABI4* lines did not show a pronounced late-flowering phenotype (Fig. 1C, F). Altogether, the early-flowering phenotype of the *abi4* mutant is consistent with other reports (Foyer *et al.*, 2012; Matsoukas *et al.*, 2013).

### Flowering time regulation-related gene expression analysis

**Fig. 2.**
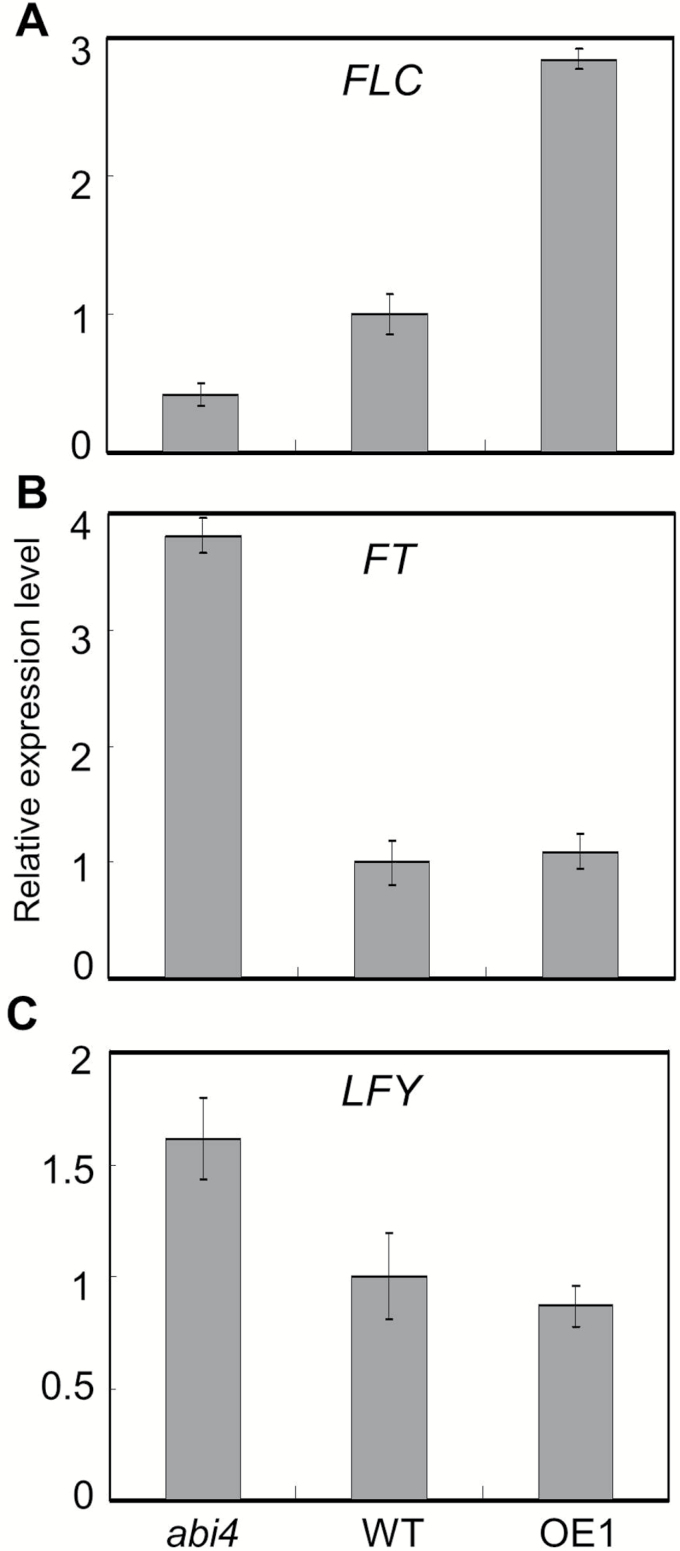
Expression analysis of the ﬂowering-time-related genes *FLC*, *FT*, and *LFY* in a*bi4*, WT, and *OE-ABI4* plants. Gene expression was detected by qRT–PCR, and three replications were performed. Primers used in the qRT–PCR assay are listed in Supplementary Table S1 at *JXB* online. (A) The *FLC* transcription level in *abi4* is decreased but is increased in *OE-ABI4* plants. (B) The *FT* expression level in *abi4* is increased. (C) The *LFY* transcription level in *abi4* is increased but is decreased in *OE-ABI4* plants.

To explore further the molecular mechanisms through which ABI4 controls floral transition, the transcription levels of some flowering time regulation-related genes were investigated in *abi4* mutant and *OE-ABI4* plants. It has been demonstrated that transcription factor FLC is the key repressor in floral transition and links the diverse flowering time regulation pathways (He, 2012). In order to explore whether the flowering phenotypes observed for the *abi4* mutant and *OE-ABI4* are correlated with the change of the expression of *FLC*, we examined the *FLC* transcript levels in these genotypes by qRT–PCR. The level of *FLC* transcript in the *abi4* mutant plants was signiﬁcantly lower, while in *OE-ABI4* plants it was significantly higher than that in the WT plants (Fig. 2A).

A previous study revealed that FLC directly represses the flowering identity gene *FT* (Searle *et al.*, 2006); thus, the *FT* expression level was also determined. Consistent with the trends in *FLC* transcription levels, the expression of *FT* also showed significant changes (Fig. 2B). In the *abi4* mutant, *FLC* expression was decreased compared with the WT (Fig. 2A), and, accordingly, *FT* transcription was significantly increased. There was no obvious change in its level in *OE-ABI4* (Fig. 2B). Furthermore, GA promotes flowering through inducing transcription of another floral identity gene. *LFY* (Blazquez *et al.*, 1998; Achard *et al.*, 2004). In a previous study, we found that ABI4 negatively regulates GA biogenesis (Shu *et al.*, 2013) and thus the *LFY* mRNA level in *abi4* and *OE-ABI4* was examined. Our result revealed that the *LFY* expression level in the *abi4* mutant was significantly increased compared with the WT (Fig. 2C). Together, the changes in the transcription levels of these three key genes were consistent with the phenotype analysis. They suggest that the flowering phenotype of both genotypes (*abi4* and *OE-ABI4*) may result from changes in the transcription levels of *FLC*, *FT*, and *LFY.*

Furthermore, to dissect the relationship between ABA and flowering control, we further detected the effect of exogenous ABA on *ABI4* transcription. The results showed that the *ABI4* expression level was strongly induced by treatment with exogenous ABA (Supplementary Fig. S2 at *JXB* online). This is in fact consistent with previous studies (Söderman *et al.*, 2000; Bossi *et al.*, 2009).

### ABI4 directly binds to the *FLC* promoter *in vivo* and *in vitro*

Previous studies have demonstrated that ABI4 binds to the CCAC motifs in some promoters to regulate the transcription of its targets genes directly (Koussevitzky *et al.*, 2007; Bossi *et al.*, 2009). We next investigated whether ABI4 directly binds to the promoters of *FLC*, *FT*, and/or *LFY in vivo* (ChIP-qPCR) and *in vitro* (EMSA).

**Fig. 3.**
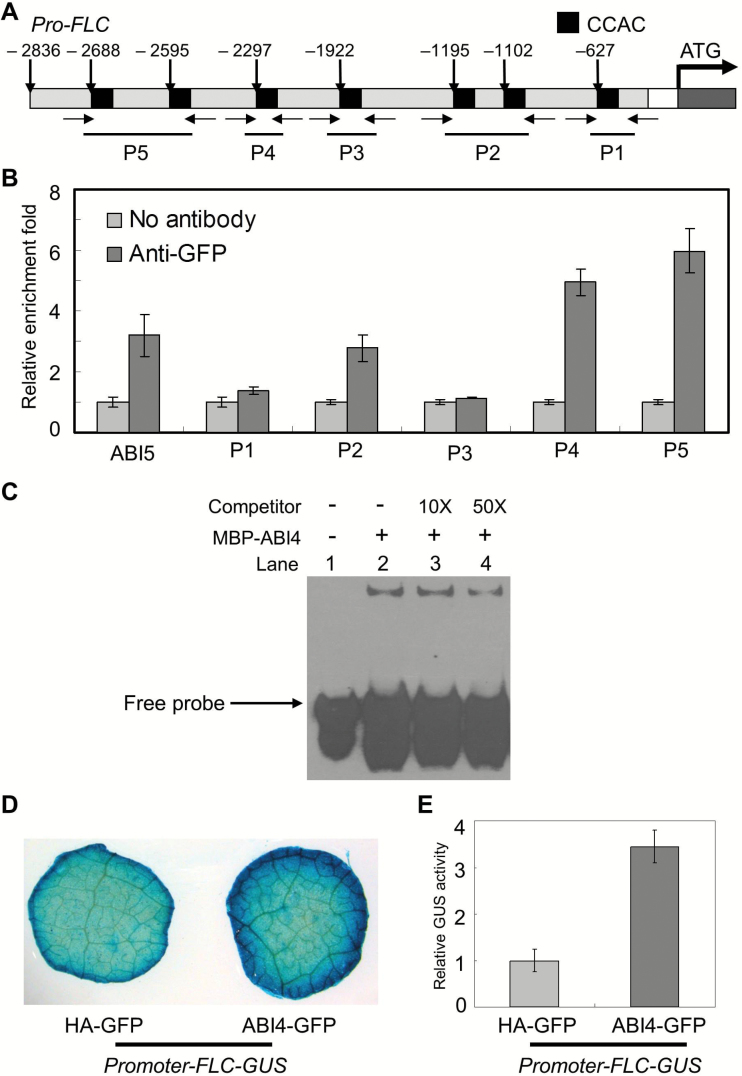
ABI4 activates *FLC* transcription by directly binding to its promoter. (A) The *FLC* promoter was analysed. Fragments located upstream of ATG were used as the promoter region. (B) ChIP-qPCR assays were performed using different specific primers corresponding to the *FLC* promoter regions. The *ABI5* promoter was used as a positive control and the *TUB4* gene was employed as an internal control. Primers used in the ChIP-qPCR assays are indicated by arrows and are presented in Supplementary Table S1 at *JXB* online. (C) EMSA results revealed that ABI4 directly interacts with the *FLC* promoter *in vitro*. The P4 fragment (A) was chosen as the probe (60bp). The P4 60bp biotin-labelled *FLC* promoter fragment is shown in the presence (lanes 2–4) or absence (lane 1) of recombinant MBP–ABI4. Non-labelled FLC promoter fragment competitors were used at a 10 (lane 3) and 50 (lane 4) molar excess. The arrow indicates the free probes. (D) Tobacco transient expression assay showed that ABI4 promotes *FLC* transcription *in vivo*. Representative GUS staining images of *N. benthamiana* leaves are shown. (E) Quantitative analysis of relative GUS activity is shown. Three biological repeats of each experiment were performed and a similar trend was seen.

We initially explored the promoters of these three genes, as shown in Fig. 3A. Seven CCAC elements were detected in the *FLC* promoter fragment. We then performed ChIP-qPCR assays with the *OE-ABI4* lines to test whether ABI4 directly binds to this promoter *in vivo*. The results revealed that the DNA fragments of the *FLC* promoter were enriched in the chromatin-immunoprecipitated DNA using the anti-GFP antibody, particularly the P4 and P5 regions, which are far from the *FLC* start codon (Fig. 3B). In addition, because a previous study demonstrated that ABI4 binds to the *ABI5* promoter directly (Bossi *et al.*, 2009), a DNA fragment of the *ABI5* promoter was used as a positive control. The sequences of the *ABI5* promoter were dissected, and the fragment used is highlighted in Supplementary Fig. S3 at *JXB* online and the primer sequences are shown in Supplementary Table S1. During this analysis, two independent transgenic lines (OE1 and OE2) were employed, and similar results were obtained. However, we did not detect the enrichment of the *FT* and *LFY* promoter fragments, although there were four and five CCAC *cis*-elements in their promoters, respectively.

Based on the ChIP-qPCR results, an EMSA was employed to confirm further the interaction between the transcription factor ABI4 and the *FLC* promoter *in vitro*. The recombinant ABI4 fusion protein was expressed and purified from *E. coli*, and the P4 fragment (Fig. 3A) was chosen as a probe for this assay. The results revealed that the mobility rate of the P4 fragment was significantly delayed in the presence of ABI4 protein (Fig. 3C, lane 2). Further, the cold-competitor probes (excess of unlabelled fragments) were sufficient to compete for the ABI4 binding activity (Fig. 3C, lanes 3–4). Taken together, these EMSA results demonstrated that ABI4 indeed directly binds to the *FLC* promoter fragments, which is consistent with the ChIP-qPCR evidence.

### ABI4 activates *FLC* transcription *in vivo*

Combined with the qRT-PCR data (Fig. 2A), the ChIP-qPCR (Fig. 3B) and EMSA (Fig. 3C) results indicate that ABI4 may activate *FLC* transcription by directly binding to its promoter. To explore the effect of ABI4 on *FLC* expression directly, we made use of the transient expression system to investigate whether ABI4 activates the expression of *FLC in vivo.*

The reporter plasmid *Promoter-FLC-GUS* and the effector plasmid *pCanG-ABI4-GFP* were constructed separately. Normal levels of GUS activity was detected when the *Promoter-FLC-GUS* construct combined with *pCanG-HA-GFP* (Fig. 3D, E). Subsequently, when the control vector *pCanG-HA-GFP* was substituted for an equal amount of the effector *pCanG-ABI4-GFP*, GUS activity increased significantly (Fig. 3D, E). These results suggest that ABI4 has an activation effect on *FLC* transcription *in vivo*. To study further the effect of the CCAC motifs on this activation effect, we mutated the key CCAC elements (altered to CCAA) in the *FLC* promoter. The P4 and P5 fragments were chosen as they showed the highest binding activity for ABI4 protein, revealed by ChIP-qPCR and EMSA (Fig. 3). Subsequently, the constructs *Pro-FLC (m1)-GUS* and *Pro-FLC (m2)-GUS* were generated (Supplementary Fig. S4A at *JXB* online). Using the transient expression system, the results revealed that the activation effect of ABI4 on *FLC* transcription was impaired in the presence of the mutated promoter constructs (Supplementary Fig. S4B–E). Altogether, combined with the ChIP-qPCR and EMSA results, the transient expression system analysis demonstrated that ABI4 directly promotes *FLC* expression, and this effect is dependent on some key CCAC elements in the *FLC* promoter.

### *ABI4* acts upstream of *FLC* genetically to regulate flowering time

The phenotypic analysis, and biochemical and molecular evidence demonstrated that the transcription factor ABI4 negatively regulates flowering time through activation of *FLC* expression. To confirm this conclusion further, the genetic relationship between *ABI4* and *FLC* was explored.

**Fig. 4.**
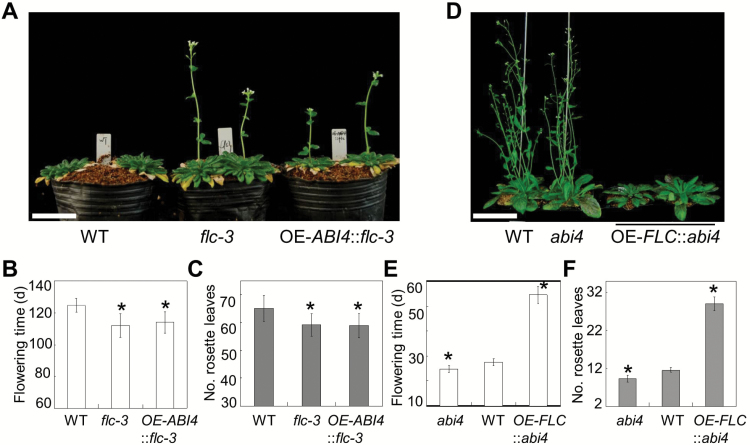
The genetic analysis of *ABI4* and *FLC*. *OE-ABI4::flc-3* and *OE-FLC::abi4* were generated by genetic crossing or transformation separately, and then the flowering time phenotype of these genotypes was examined. Bar=1cm. (A) Representative images of WT, *flc-3*, and *OE-ABI4::flc-3* plants grown under SD conditions (110 d old). (B) Flowering time scored as the days from germination to bolting of WT, *flc-3*, and *OE-ABI4::flc-3* genotypes under SD conditions. *n* ≥15; error bars indicate the SE. (C) Flowering time scored as the number of rosette leaves at flowering of WT, *flc-3*, and *OE-ABI4::flc-3* genotypes under SD conditions. *n* ≥15; error bars indicate the SE. (D) Representative WT, *abi4*, and *OE-FLC::abi4* plants grown under LD conditions (29 d old). (E) Flowering time scored as the days from germination to bolting of WT, *abi4*, and *OE-FLC::abi4* genotypes under SD conditions. *n* ≥15; error bars indicate the SE. (F) Flowering time scored as the number of rosette leaves at flowering of WT, *abi4*, and *OE-FLC::abi4* genotypes under SD conditions. *n* ≥15; error bars indicate the SE. An asterisk (*) indicates a significant difference at the *P*<0.05 level by Student’s *t*-test analysis.

The *OE-ABI4* construct was introduced into the *flc-3* mutant background by genetic crossing, and the flowering phenotypes of *flc-3* and *OE-ABI4::flc-3* were examined. In our experimental conditions, we detect the early-flowering phenotype of *flc-3* mutant plants, and overexpression of *ABI4* did not change the early-flowering phenotype of *flc-3* in terms of the number of rosette leaves and the days to flowering (Fig. 4A–C), although *OE-ABI4* in the WT background significantly delayed the floral transition (Fig. 1). On the other hand, we also generated transgenic overexpression of *FLC* in the *abi4* mutant background (*OE-FLC::abi4*) by transgenic assay, and examined the floral phenotype of *abi4* and *OE-FLC::abi4*. The results showed that overexpression of *FLC* significantly changed the early-flowering phenotype of *abi4* (Fig. 4D–F), mimicking the clearly late-flowering phenotype of *OE-FLC::WT.* Together, the genetic analysis indicated that *ABI4* acts upstream of *FLC* in the same genetic pathway.

## Discussion

The activation effect of the phytohormone GA on plant floral transition has been thoroughly investigated in the past decades. In contrast, the mechanisms by which ABA affects plant flowering time are not fully understood. Our study, using phenotypic, biochemical, and genetic analysis, has demonstrated that the transcription factor ABI4, a positive regulator of the ABA signalling pathway, negatively regulates flowering through activating *FLC* expression. However, we believe that this is the tip of an iceberg, and further details of the regulatory mechanisms of ABA on plant floral transition will be discovered in the near future.

### ABI4 negatively regulates flowering time

During the past few decades, four distinct pathways that affect plant flowering time in response to internal signals and external factors were comprehensively described in the model plant *Arabidopsis* (He, 2012, 2009). In the agronomic research field, the flowering time (heading date) is a critical factor in determining adaptation to different cultivation areas and cropping seasons (Yamamoto *et al.*, 2000). As a consequence, flowering time regulation mechanisms have attracted increasing attention.

A number of studies have demonstrated that some mutants involved in the ABA signalling pathway reveal the flowering phenotype, including *abi5* (Wang *et al.*, 2013), *CmMYB2-RNAi* plants (Shan *et al.*, 2012), and *abi4* (Foyer *et al.*, 2012; Matsoukas *et al.*, 2013). Generally, the ABA-insensitive mutants have the early-flowering phenotype, and the ABA-sensitive mutants have the late-flowering phenotype. Additionally, ABA biogenesis pathway mutants such as *aba2* also reveal the flowering phenotype as a consequence of alteration of endogenous ABA biogenesis (Domagalska *et al.*, 2010). This is in contrast to the GA-deficient mutant *ga1-3* which has a drastically late-flowering phenotype as a consequence of a large reduction in the internal GA level (Blazquez *et al.*, 1998). Although the molecular mechanisms by which GA promotes flowering are well described, the mechanism by which ABA regulates flowering has been unclear, especially for the effect of ABI4 on floral regulation.

Prior to our study, two independent groups had reported that mutations at the *ABI4* locus promote plant flowering (Foyer *et al.*, 2012; Matsoukas *et al.*, 2013). The mutant alleles in these studies are different. In the study by Foyer *et al.* (2012), the *abi4* mutant originated from sugar-insensitive (*sis*) mutant screening, and is a point mutation generated by ethylmethane sulphonate (EMS)-induced mutagenesis. It has also been called *sugar-insensitive 5* (*sis5*) (Laby *et al.*, 2000; Kerchev *et al.*, 2011; Foyer *et al.*, 2012). In the study reported by Matsoukas *et al.* (2013), the *abi4* mutant resulted from a T-DNA insertion line occurring at the *ABI4* locus. Additionally it has been called *glucose insensitive 6* (*gin6*) (Arenas-Huertero *et al.*, 2000; Matsoukas *et al.*, 2013). The *abi4* mutant used in our study arose from a point mutation at the *ABI4* locus and originated from Finkelstein’s group (Finkelstein, 1994; Finkelstein *et al.*, 1998). It is different from the allelic mutants, *sis5* and *gin6*. The fact that all three allelic mutants show a similar phenotype strongly suggests that mutations in the *ABI4* locus indeed are responsible for the altered flowering phenotypes of *abi4* and *OE-ABI4* plants (Fig. 1).

As described above, *ABI4* was also identified from the screening of sugar- or glucose-insensitive mutants, and its alternative names are *SIS5* and *GIN6*. Actually, sugar indeed affects flowering time: it promotes floral transition, but high concentrations of sugar remarkably delay flowering (Zhou *et al.*, 1998; Ohto *et al.*, 2001). The detailed mechanisms underlying this delayed effect resulted from the delayed activation of *LFY* transcription (Ohto *et al.*, 2001). However, the more precise mechanisms through which sugar content and/or signalling regulate plant floral transition need further investigation. Furthermore, it is noted that the transgenic *OE-ABI4* had the late flowering phenotype in terms of days to flowering, but not in terms of the number of rosette leaves (Fig. 1). This apparent inconsistency has been reported previously (Mai *et al.*, 2011). It may be a consequence of the very weak growth of *OE-ABI4* plants (Shu *et al.*, 2013). Actually, a previous study has also demonstrated that *OE-ABI4* seedlings are weak in terms of root length and shoot growth (Shkolnik-Inbar and Bar-Zvi, 2010), which is consistent with our investigation.

### ABI4 is a novel factor in the ABA signalling pathway which inhibits floral transition

The promotion effect of GA on plant floral transition has been well documented (Andres and Coupland, 2012; He, 2009, 2012). In contrast, the mechanism by which ABA affects flowering has been elusive (Wang *et al.*, 2013). A recent study demonstrated that application of exogenous ABA delays flowering time, and the bZIP transcription factor genes *ABI5*, *ABF1*, *ABF3*, and *ABF4* play negative roles in ABA-mediated inhibition of floral transition (Wang *et al.*, 2013). These genes are key components in the ABA signalling transduction pathway, indicating that they have important functions through which ABA affects floral transition.

ABI4 is another versatile factor which promotes ABA signalling, to regulate diverse physiological processes including seed dormancy, seed germination, lateral root initiation, and cross-talk between many hormones including ABA and GA, and ABA and auxin (Finkelstein *et al.*, 1998; Shkolnik-Inbar and Bar-Zvi, 2010; Foyer *et al.*, 2012; Shu *et al.*, 2013). In addition to these, our study suggests other possible roles for ABI4 in the regulation of plant floral transition. As well as the phenotypic description, and genetic and biochemical analysis, we described the mechanism by which ABI4 directly activates transcription of the key floral repressor *FLC*, and negatively regulates floral transition. Our findings suggest that just like bZIP transcription factors, ABI4 is a novel regulator involved in the ABA signalling pathway and inhibits plant flowering.

The negative effect of ABA on floral transition has been investigated prior to our studies (Domagalska *et al.*, 2010; Wang *et al.*, 2013). Further, the inducing effect of ABA on *ABI4* transcription was detected in the present study (Supplementary Fig. S2 at *JXB* online) and in previous studies (Söderman *et al.*, 2000; Bossi *et al.*, 2009). Combined with the present available evidence, we suggest that ABA inhibits the floral transition by activating *FLC* transcription through ABI4, at least partially. In addition, it was recently reported that ABA is required for the drought-escape response through positively regulating plant flowering (Riboni *et al.*, 2013). This is logical, as under normal growth conditions the endogenous ABA level will negatively regulates the floral transition, in contrast to the effect of GA. However, the environmental stress of drought elevates ABA levels, promoting flowering, and allowing the plant to complete its life cycle.

### *FLC* is the target of both ABI4 and ABI5

Extensive studies demonstrated that FLC is the key integrator which links the four flowering regulation pathways and inhibits plant floral transition (Koornneef *et al.*, 1998; Komeda, 2004; Andres and Coupland, 2012; Song *et al.*, 2013). Therefore, the mechanisms regulating *FLC* at the transcription level are of the utmost importance for controlling floral transition. A previous study demonstrated that the pattern of *FLC* expression is associated with epigenetic modification and changes in chromatin structure (Dennis and Peacock, 2007). Many factors are involved in this, including acetylation and methylation, and these modifications usually result in a protein complex to regulate *FLC* transcription collaboratively (Kim and Michaels, 2006; Deal *et al.*, 2007).

The SUPPRESSOR OF FRIGIDA 4 (SUF4)-mediated transcription factor complex negatively regulates flowering through promoting *FLC* expression directly; thus, the *suf4* mutant has the early-flowering phenotype (Choi *et al.*, 2011). We found that *FLC* expression is also directly regulated by the transcription factor ABI4 (Fig. 3). Another transcription factor, ABI5, involved in the ABA signalling pathway has also been reported to regulate *FLC* transcription directly (Wang *et al.*, 2013). Together, the evidence suggests that a number of transcription factors control *FLC* expression. FLC appears to be the target of both ABI4 and ABI5 concurrently. In line with this, Reeves *et al.* (2011) demonstrated that ABI4 and ABI5 share some target genes. Furthermore, a recent study demonstrated that *Diacylglycerol acyltransferase1* (*DGAT1*), encoding the rate-limiting enzyme in the triacylglycerol biosynthesis pathway (Kong *et al.*, 2013), is also regulated by ABI4 and ABI5 concurrently. It seems, therefore, that ABI5 may be an accessory factor with ABI4 in the regulation of a number of genes.

*FLC* is the target of both the the transcription factors ABI4 and ABI5; thus an interesting question arises. How do the plant discriminate between the roles of ABI4 and ABI5 in flowering time regulation? Combined with a previous study, we know that both transcription factors bind to the *FLC* promoter through different motifs. ABI4 binds the CCAC motif (Fig. 3), while ABI5 binds the *FLC* promoter through ABRE (abscisic acid-responsive element) or ABRE-like elements (CATGCG) (Wang *et al.*, 2013). Thus we speculated that the flanking sequences of the CCAC and/or CATGCG motifs may possess some cryptic and elusive effects affecting the interaction between the *FLC* promoter and ABI5/ABI4. Consequently, further bioinformatics analysis is needed to deepen our understanding of the overlap and/or distinct roles of ABI5 and ABI4 in the control of flowering.

**Fig. 5.**
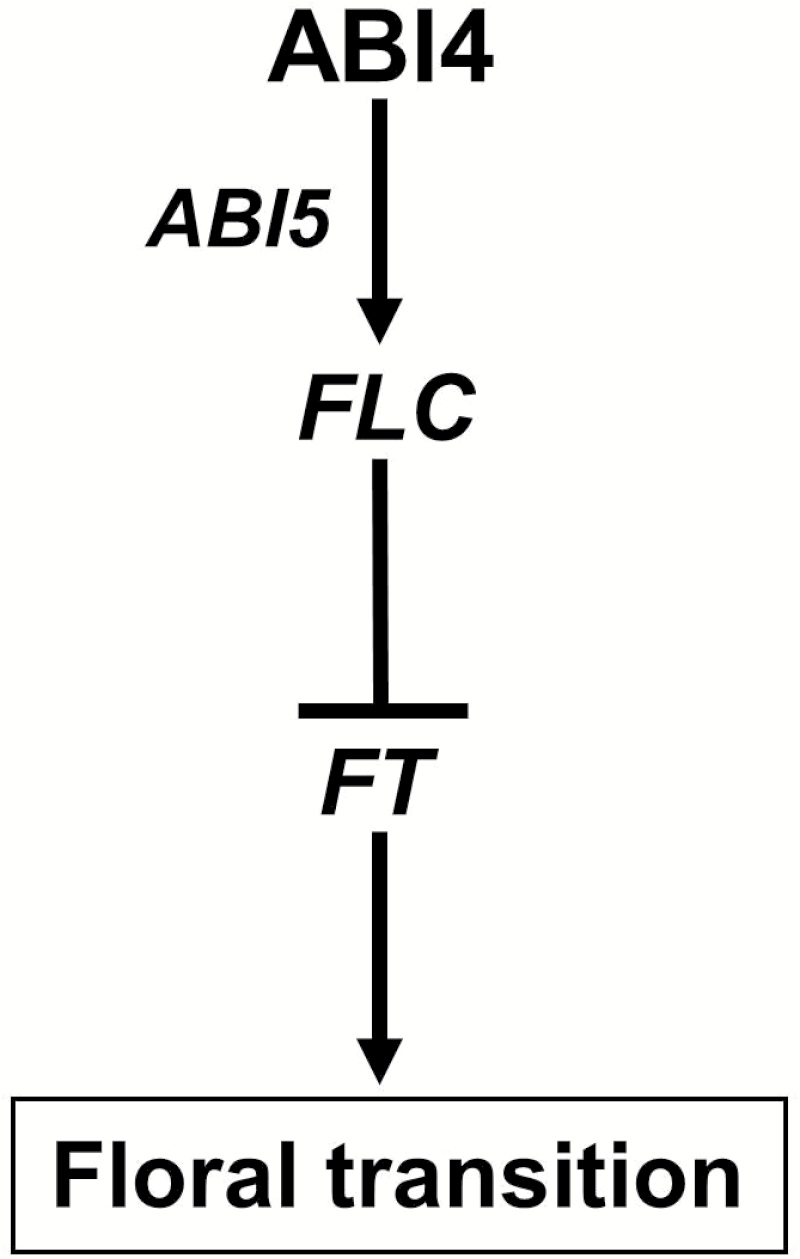
A proposed working model in which ABA inhibits floral transition through activating *FLC* transcription by ABI4 and ABI5. Transcription factors ABI4 and/or ABI5 involved in the ABA signalling pathway directly regulate *FLC* expression to control plant floral transition precisely. Because ABI4 directly binds to the *ABI5* promoter (Bossi *et al.*, 2009), ABI4 may also activate *FLC* transcription by increasing *ABI5* expression. It should be noted that ABI4 could bind to its own promoter region and activates its own transcription.

Taking our findings together, we propose a working model illustrated in Fig. 5. In the ABA signalling pathway, the transcription factors ABI4 and/or ABI5 regulate *FLC* expression directly to control plant floral transition precisely. Because ABI4 also binds directly to the *ABI5* promoter and activates its transcription (Bossi *et al.*, 2009), ABI4 may also activate *FLC* transcription through increasing *ABI5* expression. Interesting, it is noted that the model allows for the possibility that other mutants involved in the ABA signalling pathway, such as *abi3*, may regulate the flowering time, which should be the focus of future investigations.

## Supplementary data

Supplementary data are available at *JXB* online.

Figure S1. Late-flowering phenotype of *OE-ABI4* under long-day conditions.

Figure S2. ABA induces *ABI4* transcription.

Figure S3. The *ABI5* promoter was dissected.

Figure S4. ABI4 promotes *FLC* transcription in a CCAC-dependent manner.

Table S1. The primers used in this study.

Supplementary Data
