# Supplementary material for: ABSCISIC ACID-INSENSITIVE 4 negatively regulates flowering through directly promoting Arabidopsis FLOWERING LOCUS C transcription
Source: J Exp Bot. 2015 Oct 27;67(1):195–205. doi: 10.1093/jxb/erv459 (PMC4682436; doi:10.1093/jxb/erv459)
Supplement: Supplementary Data [file supp_67_1_195__index.html]

ABSCISIC ACID-INSENSITIVE 4 negatively regulates flowering through directly promoting Arabidopsis FLOWERING LOCUS C transcription — ABSCISIC ACID-INSENSITIVE 4 negatively regulates flowering through directly promoting Arabidopsis FLOWERING LOCUS C transcription — Supplementary Data 

# ABSCISIC ACID-INSENSITIVE 4 negatively regulates flowering through directly promoting Arabidopsis *FLOWERING LOCUS C* transcription

## Supplementary Data

Data files

- Supplementary Data - Supplementary Data
