# Supplementary material for: ABSCISIC ACID-INSENSITIVE 4 negatively regulates flowering through directly promoting Arabidopsis FLOWERING LOCUS C transcription
Source: J Exp Bot. 2015 Oct 27;67(1):195–205. doi: 10.1093/jxb/erv459 (PMC4682436; doi:10.1093/jxb/erv459)

ABI4 Negatively regulates flowering through directly promoting Arabidopsis FLOWERING LOCUS C transcription. *Kai Shu, Qian Chen, Yao Rong Wu, Rui Jun Liu, Hua Wei Zhang, Sheng Fu Wang, San Yuan Tang, Wen Yu Yang, and Qi Xie*

Supplementary Table S1. Primers used in this study.

**For the several transgenic constructs**	
pCanG-ABI4-GFP-Fw (XbaI)	tctagaATGGACCCTTTAGCTTCCCA
pCanG-ABI4-GFP-Rev (XbaI)	tctaga ATAGAATTCCCCCAAGATGG
ProFLC-GUS-Fw (Hind III)	aagcttTACTGCTACAGATAGATGCAG
ProFLC-GUS-Rev (BamHI)	ggatccATGGACTTTCAAACGATGGG
ProFLC (m1)-GUS-Fw	tatatttgctccaatttatctgac
ProFLC (m1)-GUS-Rev	gtcagataaattggagcaaatata
ProFLC (m2)-GUS-Fw	ctgccaaacaattaatagtaaag
ProFLC (m2)-GUS-Rev	ctttactattaattgtttggcag
pCanG-FLC-GFP-Fw (XbaI)	tctagaATGGGAAGAAAAAAACTAGAAATC
pCanG-FLC-GFP-Rev (KpnI)	ggtaccATTAAGTAGTGGGAGAGTC
**For the qRT-PCR**	
qrt-FLC-Fw	CCGAACTCATGTTGAAGCTTGTTGAG
qrt-FLC-Rev	CGGAGATTTGTCCAGCAGGTG
qrt-FT-Fw	CCTCAGGAACTTCTATACTTTGGTTATGG
qrt-FT-Rev	CTGTTTGCCTGCCAAGCTGTC
qrt-LFY-Fw	CATTGGTTGGTGACTGATAT
qrt-LFY-Rev	GCGAGTGTTGAAGTTCTG
qRT-18S-Fw	GTTGATCCTGCCAGTAGT
qRT-18S-Rev	ATCCGAGTAGTAGTTACCATC
**For ChIP-qPCR assay**	
qrt-ChIP-ABI5-1-Fw	CCTGTCTAAGTTAGCATTCCATTG
qrt-ChIP-ABI5-1-Rev	GGTTCTCCTCCTTCACATAGTT
qrt-ChIP-FLC-P1-Fw	AGGAAAACAAGCTGATACAAGCA
qrt-ChIP-FLC-P1-Rev	AACCACTCGCCTACGTCATC
qrt-ChIP-FLC-P2-Fw	GTGTGGCTCCAATAGAAAAGTT
qrt-ChIP-FLC-P2-Rev	GAGGCTATATCGAGTAACAAGACG
qrt-ChIP-FLC-P3-Fw	GCATATGCAATAGCGAGCAGTG
qrt-ChIP-FLC-P3-Rev	CTATCAGGGGCGGACTCACG
qrt-ChIP-FLC-P4-Fw	CAGAAAAGGGCAAGGAGGTG
qrt-ChIP-FLC-P4-Rev	CTTTCTTTTGGTATGGTGTCAATAT
qrt-ChIP-FLC-P5-Fw	TTGCTCCACTTTATCTGACTAGTTG
qrt-ChIP-FLC-P5-Rev	CAAGGCATCTCTATGTTTTCAAGTT
TUB4-Fw	CGAGAGGATCACAGCAATACAG
TUB4-Rev	GGATCCATTCCACAAAGTAGGA

**For mutants verification**	
flc-3 genotyping-Fw	TTGCATCACTCTCGTTTACCC
flc-3 genotyping-Rev	GCGTCACAGAGAACAGAAAGC
LBb1.3	ATTTTGCCGATTTCGGAAC
dCAPs-*abi4*-Fw （CS8104）	GCCACCGTAGGAGGAGGATC
dCAPs-*abi4*-Rev （CS8104）	TGTTGGAATTGTCCCATCTGGA
**For EMSA assay**	
pMalC2-ABI4-MBP-Fw (Bam HI)	ggattcATGGACCCTTTAGCTTCCC
pMalC2-ABI4-MBP-Rev (Xba I)	tctagaTTAATAGAATTCCCCCAAGATG
Probe-P4-Fw	taaaaattcgatatttttattatctgccacacaattaatagtaaagtttttatattgacacc
Probe-P4-Rev	ggtgtcaatataaaaactttactattaattgtgtggcagataataaaaatatcgaattttta

Shu et al., Fig. S1

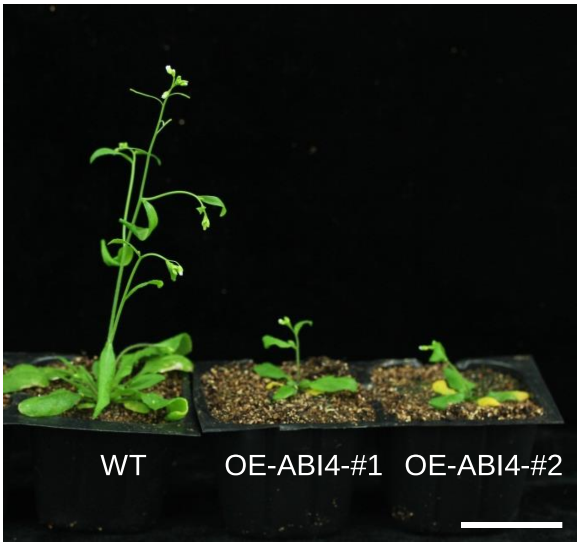

Shu et al., Fig. S2

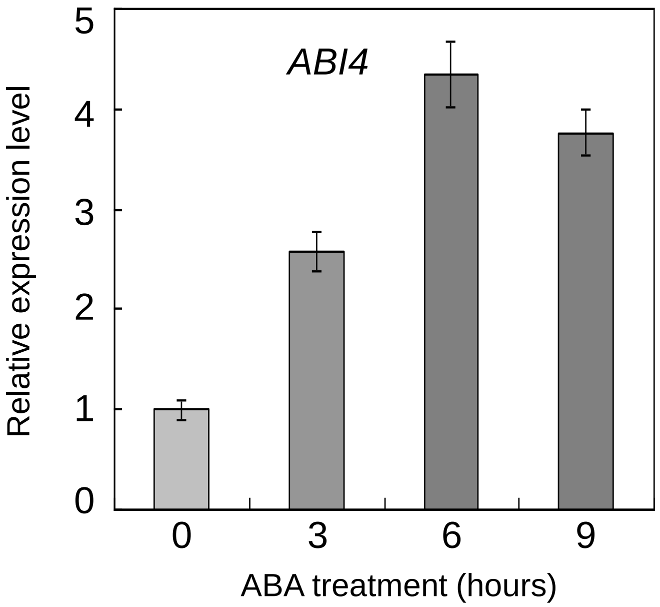

Shu et al., Fig. S3

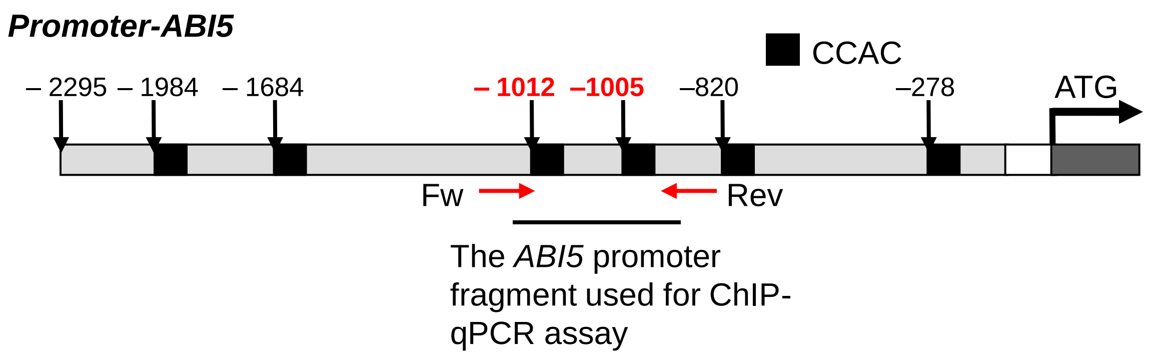

Shu et al., Fig. S4

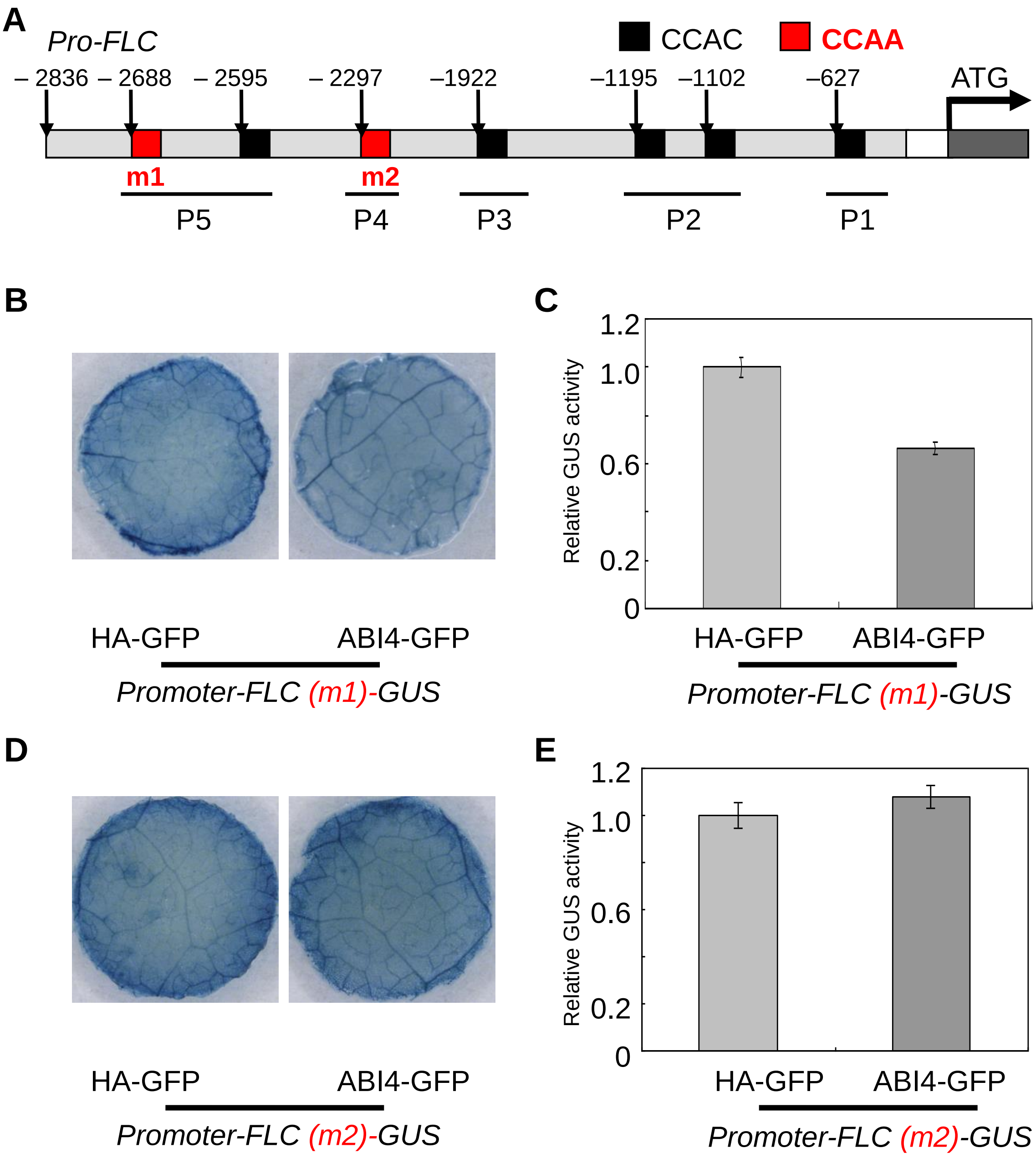

Supplement: Supplementary Data [file supp_erv459_Supplementary_data.pdf]
